# Workgroup Report: Drinking-Water Nitrate and Health—Recent Findings and Research Needs

**DOI:** 10.1289/ehp.8043

**Published:** 2005-06-23

**Authors:** Mary H. Ward, Theo M. deKok, Patrick Levallois, Jean Brender, Gabriel Gulis, Bernard T. Nolan, James VanDerslice

**Affiliations:** 1 Division of Cancer Epidemiology and Genetics, National Cancer Institute, National Institutes of Health, Department of Health and Human Services, Bethesda, Maryland, USA; 2 Department of Health Risk Analysis and Toxicology, University of Maastricht, the Netherlands; 3 Institut National de Santé Publique du Québec and Unité de recherche en santé publique, Centre Hospitalier Universitaire de Québec, Québec, Canada; 4 Department of Health Services Research, Texas State University, San Marcos, Texas, USA; 5 Department of Health Promotion Research, Southern Denmark University and Department of Public Health, University of Trnava, Slovakia; 6 U.S. Geological Survey, Reston, Virginia, USA; 7 Washington State Department of Health, Olympia, Washington, USA

**Keywords:** adverse reproductive outcomes, methemoglobinemia, neoplasms, nitrate, nitrite, *N*-nitroso compounds, water pollution

## Abstract

Human alteration of the nitrogen cycle has resulted in steadily accumulating nitrate in our water resources. The U.S. maximum contaminant level and World Health Organization guidelines for nitrate in drinking water were promulgated to protect infants from developing methemoglobinemia, an acute condition. Some scientists have recently suggested that the regulatory limit for nitrate is overly conservative; however, they have not thoroughly considered chronic health outcomes. In August 2004, a symposium on drinking-water nitrate and health was held at the International Society for Environmental Epidemiology meeting to evaluate nitrate exposures and associated health effects in relation to the current regulatory limit. The contribution of drinking-water nitrate toward endogenous formation of *N*-nitroso compounds was evaluated with a focus toward identifying subpopulations with increased rates of nitrosation. Adverse health effects may be the result of a complex interaction of the amount of nitrate ingested, the concomitant ingestion of nitrosation cofactors and precursors, and specific medical conditions that increase nitrosation. Workshop participants concluded that more experimental studies are needed and that a particularly fruitful approach may be to conduct epidemiologic studies among susceptible subgroups with increased endogenous nitrosation. The few epidemiologic studies that have evaluated intake of nitrosation precursors and/or nitrosation inhibitors have observed elevated risks for colon cancer and neural tube defects associated with drinking-water nitrate concentrations below the regulatory limit. The role of drinking-water nitrate exposure as a risk factor for specific cancers, reproductive outcomes, and other chronic health effects must be studied more thoroughly before changes to the regulatory level for nitrate in drinking water can be considered.

Humans have altered the nitrogen cycle dramatically over the last half-century, and as a result, nitrate is steadily accumulating in our water resources. Globally, human nitrogen production has increased rapidly since 1950 and currently exceeds nitrogen fixed by natural sources by about 30% (Fields 2004). This figure compares with pre-1950 human inputs, which were a small fraction of the input from natural sources (Lambert and Driscoll 2003). Fertilizer is the largest contributor to anthropogenic nitrogen worldwide; other major sources include animal and human waste, nitrogen oxides from utilities and automobiles, and leguminous crops that fix atmospheric nitrogen (Fields 2004). These organic and inorganic sources of nitrogen are transformed to nitrate by mineralization, hydrolysis, and bacterial nitrification. Under reducing conditions, nitrate can be biologically transformed to nitrogen gas through denitrification. Nitrate not taken up by plants or denitrified migrates to streams and groundwater.

The U.S. Environmental Protection Agency (EPA) maximum contaminant level (MCL) for nitrate in drinking water of 10 mg/L nitrate-nitrogen (nitrate-N) (equivalent to 45 mg/L as nitrate) and the World Health Organization (WHO) guideline (WHO 2004b) of 50 mg/L as nitrate (equivalent to 11 mg/L as nitrate-N) were promulgated to protect against methemoglobinemia, or “blue baby syndrome,” to which infants are especially susceptible. The regulatory level is usually met for public water supplies, which are routinely monitored. Much less is known about private wells, which in the United States are usually required to be tested only when the well is constructed or when the property is sold. Some have suggested recently that the regulatory level for nitrate in drinking water is overly conservative (Avery 1999; L’hirondel and L’hirondel 2002). However, this discussion of the regulatory level has not thoroughly considered studies of other chronic health effects including cancer, adverse reproductive outcomes, and diabetes. Although a causal role for nitrate in these other health outcomes is not conclusive, recent studies that indicate possible adverse effects at nitrate levels below the MCL are of concern (Brender et al. 2004b; DeRoos et al. 2003; Ward et al. 1996; Weyer et al. 2001).

In recognition of the widespread contamination of drinking-water sources by nitrate and the potential for health effects in addition to methemoglobinemia, a symposium titled “Drinking Water Nitrate and Health: Recent Findings and Research Needs” took place at the annual meeting of the International Society for Environmental Epidemiology (1–4 August 2004, New York, New York, USA). Invited experts presented results from recent unpublished studies and summarized the state of knowledge on exposure and health effects of drinking-water nitrate, with a focus on cancer and adverse reproductive outcomes. This article summarizes the symposium discussions and recommends promising areas for future research. Specifically, we discuss the epidemiologic evidence for drinking-water nitrate and risk of specific cancers, adverse reproductive outcomes, and other health outcomes in the context of the current regulatory limit for nitrate in drinking water.

## Nitrate Levels in Groundwater and Water Supplies

**Figure 1:**
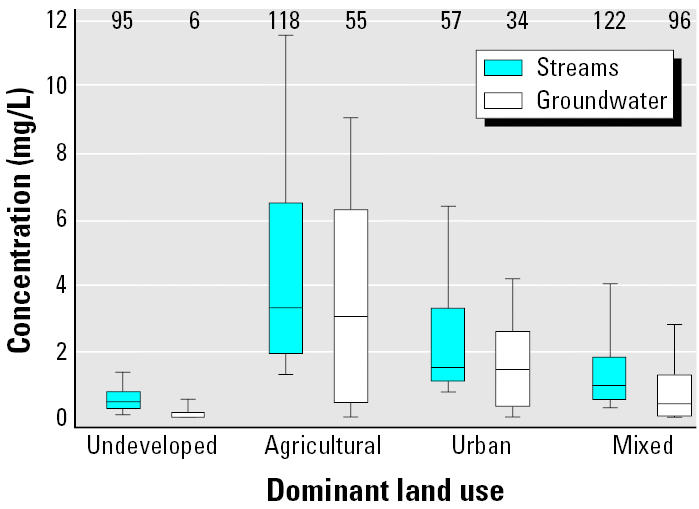
Interquartile range of total nitrogen in streams and nitrate-N in groundwater in agricultural, urban, and mixed land use, and undeveloped areas of the United States. Upper bound of bar represents 90th percentile and lower bound represents 10th percentile. Along the top of the graph are the number of stream sampling stations and groundwater networks (group of wells in an aquifer).

Nitrate is the most common chemical contaminant in the world’s groundwater aquifers (Spalding and Exner 1993). An estimated 42% of the U.S. population uses groundwater as their drinking-water supply (Hutson et al. 2004). In the United States, total nitrogen in streams and nitrate in groundwater are highest in agricultural areas, followed by urban areas and areas with mixed land use (Figure 1). The most recent data indicate that about 22% of domestic wells in agricultural areas of the United States exceeded the MCL (U.S. Geological Survey, unpublished data). In contrast, 3% of public supply wells in major aquifers (typical sources for public water supplies) exceed the MCL (U.S. Geological Survey, unpublished data).

The exposure picture is similar in the European Union. Public water supplies are largely below the WHO guideline; however, in some countries, private wells in rural areas have elevated nitrate concentrations reaching 10–15 times the recommended level (European Environment Agency 2003). Overall, nitrate levels exceeded the guideline in about one-third of the groundwater bodies for which data were available (European Environment Agency 2003). Several eastern European countries report high levels of nitrate contamination in a large proportion of private wells; for example, in Romania, 20% of 2,000 wells had nitrate levels > 23 mg/L as nitrate-N (Jedrychowski et al. 1997). Studies from other countries, including China, Botswana, Turkey, Senegal, and Mexico, report private well water levels that exceed the WHO guideline, in some instances at levels > 68 mg/L nitrate-N (WHO 2004a). Fertilizer is the main contributing factor in agricultural areas; however, nitrogen from human waste appears to be the most important source in urban areas lacking centralized water and sanitation systems. Although systematic information on nitrate levels in groundwater in other parts of the world is more limited, empirical modeling approaches have indicated that users of shallow wells in areas with high nitrogen inputs, well-drained soils, and unconsolidated rocks are most at risk of consuming high-nitrate groundwater (Nolan et al. 2002).

## Methemoglobinemia

Ingested nitrate is reduced to nitrite, which binds to hemoglobin to form methemoglobin (MetHb). Methemoglobinemia occurs when elevated levels of MetHb (exceeding about 10%) interfere with the oxygen-carrying capacity of the blood. Infants are particularly susceptible to developing methemoglobinemia for several reasons, including their increased capacity to convert nitrate to nitrite and their lower levels of the enzyme cytochrome b5 reductase, which converts MetHb back to hemoglobin. Methemoglobinemia in infants fed formula made with well water with high nitrate levels was first reported in 1945 by Comly (1945). The regulatory level for nitrate in drinking-water supplies was determined after a survey of infant methemoglobinemia case reports in the United States indicated that no cases were observed at drinking-water nitrate levels < 10 mg/L nitrate-N (Walton 1951). Because an estimated 22% of domestic wells in agricultural regions of the United States exceed the nitrate MCL (U.S. Geological Survey, unpublished data), it is likely that significant numbers of infants are given water containing > 10 mg/L nitrate-N. Nevertheless, few cases of methemoglobinemia have been reported since the MCL was promulgated.

The risk of methemoglobinemia among infants depends on many factors other than the ingestion of nitrate in drinking water. Some foods and medications contain high levels of nitrate (Sanchez-Echaniz et al. 2001). Enteric infections, potentially caused by fecal bacteria contamination in wells, may lead to the endogenous production of nitrite, as evidenced by numerous published reports of infants with diarrhea and methemoglobinemia but no apparent exposure to exogenous MetHb-forming agents (Charmandari et al. 2001; Hanukoglu and Danon 1996; Levine et al. 1998; Wennmalm et al. 1993). The consumption of antioxidants such as vitamin C appears to be a protective factor. Finally, polymorphisms in the activity of cytochrome b5 reductase may mediate the effect of ingested nitrate or endogenously produced nitrite (Gupta et al. 1999).

Studies that have examined the relationship between nitrate levels in drinking water and MetHb levels in infants have produced mixed results (U.S. EPA 1991). The few experimental studies are largely negative; however, most of these studies evaluated low levels of drinking-water nitrate and included few infants. Cofactors such as diarrhea and respiratory diseases reportedly increase MetHb levels (Shearer et al. 1972; Shuval and Gruener 1972). An epidemiologic study in South Africa (Super et al. 1981) found an increase in MetHb levels in infants fed water with nitrate > 20 mg/L nitrate-N; however, clinical methemoglobinemia was rarely found. A protective effect of vitamin C intake on MetHb was noted (Super et al. 1981). More recently, a retrospective, nested case–control study in Romania found an association between nitrate exposure from drinking water and clinical methemoglobinemia, but also some evidence of an association with diarrheal disease (Zeman et al. 2002). Gupta and colleagues (1999) found cytochrome b5 reductase activities to be higher among those consuming water with high nitrate levels, indicating a level of adaptation to the consumption of high nitrate waters.

Recently, the role of nitrate exposure alone in causing methemoglobinemia has been questioned (Avery 1999; Fewtrell 2004; Hanukoglu and Danon 1996). Clearly, we need to better understand the interaction of factors that lead to methemoglobinemia to assess the relative importance of each factor and to identify the conditions under which exposure to nitrate in drinking water poses a risk of methemoglobinemia.

## Nitrate Intake and Endogenous Formation of *N*-Nitroso Compounds

Nitrate is a precursor in the formation of *N*-nitroso compounds (NOC), a class of genotoxic compounds, most of which are animal carcinogens. In the human body, nitrate is a stable, inert compound that cannot be metabolized by human enzymes. However, the nitrate-reducing activity of commensal bacteria may convert nitrate into nitrite and other bioactive nitrogen compounds that affect physiological processes and human health. After ingestion, nitrate is readily absorbed from the upper gastrointestinal tract. Up to 25% is actively excreted in saliva, where about 20% is converted to nitrite by bacteria in the mouth (Spiegelhalter et al. 1976). This conversion can occur at other sites including the distal small intestine and the colon.

Under acidic conditions in the stomach, nitrite is protonated to nitrous acid (HNO_2_), which in turn spontaneously yields dinitrogen trioxide (N_2_O_3_), nitric oxide (NO), and nitrogen dioxide (NO_2_). NO is a bioactive compound known to play a role in vasodilatation and in defense against periodontal bacteria and other pathogens. N_2_O_3_, on the other hand, is a powerful nitrosating agent capable of donating NO^+^ to secondary and tertiary amines to form potentially carcinogenic *N*-nitrosamines (Leaf et al. 1989). Alternatively, HNO_2_ can be protonated to H_2_NO_2_, which reacts with amides to form *N*-nitrosamides. At neutral pH, nitrite can be reduced by bacterial activity to form NO, which can react with molecular oxygen to form the nitrosating compounds N_2_O_3_ and nitrogen tetroxide (N_2_O_4_). In addition to the acid-catalyzed and bacterial-catalyzed formation of nitrosating agents, inducible NO synthase activity of inflammatory cells can also produce NO (Ohshima and Bartsch 1994). Together, these three mechanisms of endogenous nitrosation account for an estimated 40–75% of the total human exposure to NOC (Tricker 1997). Other sources of human exposure include pre-formed NOC found in preserved meats and fish, beer, certain occupational exposures, and tobacco products (Tricker 1997).

Several studies support a direct relationship between nitrate intake and endogenous formation of NOC. High intake of drinking-water nitrate (above the MCL) is associated with an increased endogenous capacity to nitrosate proline (Mirvish et al. 1992; Moller et al. 1989). In addition, populations with high rates of esophageal and gastric cancer excrete high levels of *N*-nitrosoproline (Kamiyama et al. 1987; Lu et al. 1986). Nitrate intake at the acceptable daily intake level (3.67 mg/kg body weight, 0.84 mg/kg as nitrate-N) results in increased urinary excretion of NOC, particularly in combination with increased intake of dietary nitrosatable precursors (Vermeer et al. 1998). However, a Canadian population exposed to nitrate below the acceptable daily intake level showed no relationship between nitrate levels in drinking water and urinary nitrosamines (Levallois et al. 2000).

### Factors that modulate endogenous nitrosation.

Although intake of high drinking-water nitrate is consistently associated with endogenous nitrosation capacity, intake of dietary nitrate is less likely to increase nitrosation, because of the presence of nitrosation inhibitors in vegetables, the major contributors to dietary nitrate intake (Bartsch et al. 1988; National Academy of Sciences 1981). Dietary compounds that inhibit endogenous nitrosation include vitamin C, which has the capacity to reduce HNO_2_ to NO, and alphatocopherol, which can reduce nitrite to NO. Several epidemiologic studies reported no association or inverse associations between dietary nitrate intake and human cancers (Boeing 1991; Forman 1987; Ward et al. 1996), which may be because of the antioxidants and nitrosation inhibitors in nitrate-containing foods (Bartsch et al. 1988). Inhibitory effects on nitrosation have also been described with betel nut extracts, ferulic and caffeic acid, garlic, coffee, and green tea polyphenols (Stich et al. 1984). In addition, nondietary factors such as the use of mouthwashes containing chlorhexidine can influence the endogenous nitrosating capacity (van Maanen et al. 1998).

Apart from the level of nitrosating agents, the level of nitrosatable precursors in the diet, which come predominantly from meat and fish, is a crucial factor in endogenous nitrosation. Dietary intakes of red and processed meat are of particular importance in the formation of fecal NOC (Bingham 1999; Bingham et al. 1996, 2002; Cross et al. 2003; Haorah et al. 2001). Higher consumption of red meat (600 vs. 60 g/day), but not white meat, resulted in a 3-fold increase in fecal NOC levels (Bingham et al. 1996). Colon cancer incidence is most consistently associated with consumption of red meat (beef, lamb, and pork), but not with poultry and fish (Bingham 1999). Dietary supplementation of a diet low in red meat with either heme iron or inorganic iron demonstrated that heme in particular was able to stimulate endogenous nitrosation (Cross et al. 2003), thereby providing a possible explanation for the differences in colon cancer risk between red and white meat consumption. Additionally, this linkage may be stronger for processed meat than for fresh meat because of the higher NOC and NOC precursor levels in processed meat.

Endogenous nitrosation can also be stimulated by inflammatory and other medical conditions. For instance, patients with bilharzia have an increased bladder cancer risk associated with increased urinary levels of nitrite and volatile nitrosamines, most likely generated by the reaction of inflammation-derived NO with amines present in the urine (Tricker et al. 1989). Inflammatory bowel disease is also related to both increased nitro-sation and cancer risk (Lashner et al. 1988). During inflammatory bowel disease, increased inducible NO synthase activity can produce excess NO, which is oxidized to nitrogen oxides and nitrite, which in turn react with nitrosatable precursors in colonic contents to produce NOC. Indeed, ulcerative colitis patients showed increased levels of inducible NO synthase in the colonic mucosa (Kimura et al. 1998) and of NO and nitrite in the colonic lumen (Lundberg et al. 1997; Roediger et al. 1990). Increased levels of fecal NOC have been found in patients with inflammatory bowel disease and in mice with chemically induced colitis (de Kok et al. 2005; Mirvish et al. 2003).

## Health Effects Associated with Drinking-Water Nitrate

### Cancer.

NOC are potent animal carcinogens, inducing tumors at multiple organ sites including the esophagus, stomach, colon, bladder, lympatics, and hematopoietic system (Bogovski and Bogovski 1981). NOC cause tumors in every animal species tested, and it is unlikely that humans are unaffected (Lijinsky 1986). The number of well-designed epidemiologic studies with individual exposure data and information on nitrosation inhibitors and precursors are few for any single cancer site, limiting the ability to draw conclusions about cancer risk.

Most studies have been ecologic in design, linking incidence or mortality rates to drinking-water nitrate levels at the town or county level. The early studies focused on stomach cancer mortality, and most used drinking-water nitrate measurements concurrent with the period of cancer mortality. Results were mixed, with some studies showing positive associations, many showing no association, and a few showing inverse associations (Cantor 1997). Recent ecologic studies of stomach cancer in Slovakia, Spain, and Hungary with historical measurements and exposure levels near or above the MCL have found positive correlations with stomach cancer incidence or mortality (Gulis et al. 2002; Morales-Suarez-Varela et al. 1995; Sandor et al. 2001). Two studies included other cancer sites. In Slovakia, incidence of non-Hodgkin lymphoma (NHL) and colon cancer was significantly elevated among men and women exposed to public supply nitrate levels of 4.5–11.3 mg/L nitrate-N (Gulis et al. 2002); there was no association with bladder and kidney cancer incidence. In Spain, there was a positive correlation between nitrate levels in public supplies and prostate cancer mortality, but no relation with bladder and colon cancer (Morales-Suarez-Varela et al. 1995).

In the past decade, several case–control and cohort studies have evaluated historical nitrate levels in public water supplies (largely < 10 mg/L nitrate-N) and risk of several cancers (Table 1). Some studies evaluated factors affecting nitrosation, such as vitamin C intake. A cohort study of older women in Iowa (USA) (Weyer et al. 2001) found a 2.8-fold and 1.8-fold risk of bladder and ovarian cancers, respectively, associated with the highest quartile (> 2.46 mg/L nitrate-N) of the long-term average nitrate levels at the current residence. They observed significant inverse associations for uterine and rectal cancer and no significant associations for NHL, leukemia, colon, rectum, pancreas, kidney, lung, and melanoma. Case–control studies of bladder (Ward et al. 2003), brain (Ward et al. 2004), colon and rectum (De Roos et al. 2003), and pancreas cancer (Coss et al. 2004) in Iowa found no association between cancer risk and average nitrate levels over almost 30 years. Each study evaluated the interaction between nitrosation inhibitors or NOC precursors and nitrate intake from drinking water. For colon cancer, there was a significant positive interaction between 10 or more years of exposure above 5 mg/L nitrate-N and both low vitamin C and high meat intake, factors likely to increase endogenous NOC formation (De Roos et al. 2003).

A case–control study of NHL in Nebraska (USA) (Ward et al. 1996) found a significant positive association between the average nitrate level in public water supplies over about 40 years and risk among men and women. In the highest quartile of nitrate (4.0 mg/L nitrate-N), risk was elevated 2-fold. However, a recent study of NHL in Iowa with similar exposure levels found no association (Ward et al. 2004). A case–control study of NHL in Minnesota (USA) (Freedman et al. 2000) with lower levels of nitrate found an inverse association among those with the highest level (> 1.5 mg/L nitrate-N). Case–control studies in Nebraska (Ward et al. 2004) and Germany (Steindorf et al. 1994) found no association with long-term average nitrate levels in public water supplies and adult brain cancer. The Nebraska study found no evidence of an interaction with vitamin C intake. A case–cohort analysis of stomach cancer within a cohort study in the Netherlands (van Loon et al. 1998) found no association with quintiles of water nitrate intake determined from public supply levels.

Specific NOC are transplacental neurocarcinogens in animal studies. A study of childhood brain cancer measured nitrate levels in water supplies using dipstick measurements, often many years after the pregnancy (Mueller et al. 2001). Measured levels of nitrate and nitrite were not associated with risk; however, women in western Washington State, one of the three study centers, who used private wells as their drinking-water source during the pregnancy had a significantly increased risk of brain cancer in their offspring.

### Adverse reproductive outcomes.

In 1961, Schmitz described a possible relationship between high maternal MetHb levels and spontaneous abortion. Since then, at least 10 studies have examined the association between drinking-water nitrate and adverse reproductive outcomes. Table 2 summarizes these studies by location, study design, determination of water nitrate, and key findings. Few studies have been published regarding water nitrate and the outcomes of spontaneous abortions, stillbirths, premature birth, or intrauterine growth retardation. Results of these studies have been inconsistent, possibly indicating no true effect of water nitrate on reproductive outcomes at the levels evaluated in these studies. Alternatively, the inconsistencies may be due to the differing periods over which exposure was assessed, differing levels of water nitrate across studies, or differences in exposure to other cofactors.

Results of studies evaluating drinking-water nitrate and congenital malformations in offspring are also mixed (Table 2). Four studies (Arbuckle et al. 1988; Brender et al. 2004a, 2004b; Croen et al. 2001, Dorsch et al. 1984) found positive associations between drinking-water nitrate and congenital malformations, particularly malformations of the central nervous system, and specifically neural tube defects (NTDs). In each of these studies, water nitrate levels associated with increased risk of these defects were below the MCL, although the 95% confidence intervals (CIs) for some of the risk estimates were consistent with unity and varied by the source of water (groundwater, mixed, or surface). Two of these studies (Brender et al. 2004b; Croen et al. 2001) also examined dietary intake of nitrates and nitrates and NTDs and found minimal or no effect on risk. In a study of nitrosatable drug exposure and risk of NTDs (Brender et al. 2004b), drinking-water nitrates and dietary nitrites/total nitrites substantially modified the risk associated with this drug exposure during the periconceptional period; higher levels of nitrates in food or water significantly increased the risk of NTDs if women were exposed to such drugs.

### Other health outcomes.

Animal studies suggest that nitrate at high doses can competitively inhibit iodine uptake and induce hypertrophic changes in the thyroid (Bloomfield et al. 1961). In a human biomonitoring study in the Netherlands, consumption of water with nitrate levels at or above the MCL was associated with thyroid hypertrophy (van Maanen et al. 1994) and genotoxic effects (van Maanen et al. 1996). Animal studies provide evidence that NOC can damage the pancreatic beta cells (Longnecker and Daniels 2001). Three epidemiologic studies (Kostraba et al. 1992; Parslow et al. 1997; van Maanen et al. 2000) that were ecologic in design found a positive correlation between drinking-water nitrate levels below the MCL and the incidence of type I childhood diabetes, although the association observed by van Maanen was not statistically significant. Other studies have found associations between water nitrate exposure and increased blood pressure (Pomeranz et al. 2000) and acute respiratory tract infections in children (Gupta et al. 2000).

## Recommendations for Future Research

### Experimental/human biomonitoring studies.

Endogenous nitrosation in humans has been demonstrated in relation to drinking-water nitrate ingestion at levels above the MCL. However, further studies are needed to determine the extent of endogenous nitrosation at intermediate drinking-water nitrate levels (5–10 mg/L as nitrate-N) and to clarify the role of nitrate from water versus food sources. Furthermore, the role of precursors and modulators of NOC formation should be more fully investigated. These future studies should be conducted among healthy individuals as well as individuals with medical conditions that increase endogenous nitrosation.

In view of the complex kinetics of NOC formation and the organ specificity of several of these compounds (Hodgson et al. 1980; Suzuki et al. 1999), more studies are needed to evaluate the relationship between nitrate intake and formation, metabolism, and excretion of NOC. Ideally, a physiologically based pharmacokinetic model should be developed as previously recommended (National Research Council 1995) to predict exposure to NOC from all sources of nitrate exposure (exogenous and endogenous), nitrite intake, the transformation of nitrate into nitrite, and antioxidant intake. However, this will require additional data on the formation of individual NOC as well as their respective toxicologic characteristics. The results of these investigations will reveal the value of different markers of NOC exposure in future epidemiologic studies. Future studies linking NOC exposure to early markers of effect or to the actual disease will clarify the role of endogenous nitrosation and NOC exposure as etiologic factors.

Because many NOC require α -hydroxylation by CYP2E1 for bioactivation and for formation of DNA adducts, it is important to investigate the influence of polymorphisms in the gene encoding for this enzyme. One study found that specific variants in this gene are linked to increased rectum cancer risk, particularly in subjects with high intake of red and processed meat, who are exposed to increased levels of NOC (Le Marchand et al. 2002). Moreover, gene expression levels of human CYP2E1 were related to cytotoxicity and DNA damage by nitrosamines in pancreatic beta-cell lines, suggesting that such gene environment interactions are also relevant in type 1 diabetes (Lees Murdock et al. 2004). These promising lines of research point to a possible interaction between drinking-water nitrate exposure and gene expression of and/or genetic variation in CYP2E1, which may also influence the risk of several adverse health outcomes associated with nitrate exposure.

### Epidemiologic studies.

Methods must be developed and validated to improve estimates of current and historical exposure to nitrate via food and water, particularly for populations served by private wells, which are less likely to be routinely monitored. Future epidemiologic studies should integrate *a*) exposure assessment for nitrate intake from drinking water, nitrate and nitrite intake from the diet, and amines and amides from dietary and drug sources, *b*) endogenous exposure to NOC by analysis of relevant biological media (e.g., saliva, urine, feces), and *c*) reliable health risk markers (e.g., biomarkers of genotoxicity) or diagnosis of actual disease.

Future studies should include populations with well-characterized long-term exposures, including those who use private wells, which can have higher nitrate levels than public supplies. With the increasing availability of public water supply monitoring data (many U.S. states have almost 40 years of measurements), further detailed exposure assessment of populations using public supplies is also feasible. Drinking-water contaminants that may occur along with nitrate, such as agricultural pesticides, should also be evaluated. Geographic-based modeling efforts to predict the probability of high nitrate concentrations in groundwater, using information on nitrogen inputs from agricultural and urban sources (Nolan et al. 2002), is a promising approach for estimating drinking-water nitrate exposure for the population using private wells.

Additional studies of drinking-water nitrate and cancer are needed to follow up on the suggestive positive findings to date and to evaluate other cancer sites in which endogenously formed NOC may play a role. Studies of reproductive outcomes should address the exposure period most relevant for the specific outcome of interest. Maternal residential mobility between conception and birth may lead to misclassification of exposure if the water source at birth is used in studies of spontaneous abortions and congenital malformations. Studies must be of sufficient size to allow for examination of specific defects rather than groups of defects by system, because combining different defects might mask associations. More research is needed on the relation between water nitrate and the reproductive outcomes of spontaneous abortion, fetal death, premature birth, and intrauterine growth retardation.

In the design and analysis stage, future epidemiologic studies should consider factors that modulate endogenous nitrosation, as discussed above, to be able to evaluate potential interactions of water nitrate intake with these factors, thus providing stronger evidence for or against an association. In particular, studies of susceptible populations may be fruitful, and epidemiologic studies should be designed with sufficient power to evaluate risk among potentially susceptible subgroups. Such populations include patients with different forms of chronic inflammation (such as inflammatory bowel disease), patients infected with nitrate-reducing bacteria (such as in periodontal disease), those with low intake of vitamins and other known nitrosation inhibitors, or those with a history of high incidence of potentially NOC-related diseases. The people of Linxian County in China, for example, are known for their persistently low intake of several micronutrients and high risk of esophageal cancer (Blot et al. 1993). Such populations will likely benefit from preventive measures taken as a result of these investigations.

## Conclusions

Adverse health effects from drinking-water nitrates are most likely the result of a complex interaction of the amount of nitrate ingested, the concomitant ingestion of nitrosating cofactors and precursors, and medical conditions of the host that may increase nitrosation. Furthermore, these effects may be attenuated by inhibitors of endogenous nitrosation such as vitamin C and alphatocopherol. We recommend that future studies take into account such complexities in understanding the relation between drinking-water nitrates and cancer, adverse reproductive outcomes, and other health outcomes.

Several authors (Avery 1999; L’hirondel and L’hirondel 2002) have questioned the importance of nitrate in drinking water as a risk factor for methemoglobinemia and have suggested that the current nitrate standard might be safely raised to 15–20 mg/L nitrate-N with no increase in methemoglobinemia cases. A better understanding of the conditions under which nitrate in drinking water poses a risk of methemoglobinemia is clearly needed, particularly in light of recent cases of methemoglobinemia associated with well water levels between 20 and 30 mg/L nitrate-N (Knobeloch et al. 2000). Most importantly, the role of nitrate as a risk factor for cancer and adverse reproductive outcomes must be more thoroughly explored before changes to nitrate water quality standards are considered.

## Figures and Tables

**Table 1:** Analytic epidemiologic studies of drinking-water nitrate^a^ and cancer.

Reference, year, country	Study design (case–control, cohort) Regional description	Years of cancer ascertainment	Exposure description^a^	Cancer sites included	Summary of findings
Coss et al. 2004 USA	Population-based case–control		Average nitrate level in public supplies 1960–1987 (highest quartile > 2.8 mg/L); Years of exposure ≥ 7.5 and 10 mg/L	Pancreas	No significant associations with quartiles of average nitrate or number of years ≥ 7.5 or 10 mg/L
	Incidence				
	Iowa	1986–1989			
DeRoos et al. 2003 USA	Population-based case–control		Average nitrate level in public supplies 1960–1987 categorized into four levels (lowest: ≤ 1.0; highest: > 5mg/L); Years of exposure > 5 and >10 mg/L	Colon	No association with average level, years > 5 and 10 mg/L; Significantly elevated risk among subgroups with below median vitamin C intake or above median meat intake and 10 or more years > 5 mg/L
	Incidence			Rectum	
	Iowa	1986–1989			
Freedman 2000 USA	Population-based case–control		Average nitrate level in public water supplies 1947–1980 (157 towns) categorized into three levels: ≤ 0.5, > 0.5 to ≤ 1.5, > 1.5 mg/L	Non-Hodgkin lymphoma	No increase risk with increasing exposure level. OR for > 1.5 mg/L (three cases, four controls) was 0.3 (95% CI, 0.1–0.9).
	Incidence				
	Minnesota excluding four largest cities	1980–1982			
Mueller et al. 2001 USA	Population-based case–control 19 counties in San Francisco, California, area and western Washington State	1984–1990	Water source (private well, public supply) during pregnancy; dipstick measurements of nitrate and nitrite for those still living at residence during pregnancy	Childhood brain	No overall association with water source. Well use in western Washington State increased risk (OR = 2.6; 95% CI, 1.3–5.2); well use in Los Angeles inversely associated with risk (OR = 0.2; 95% CI, 0.1–0.8)
Steindorf et al. 1994 Germany	Population-based case–control		Nitrate levels in municipal supplies after 1970 (highest quartile: > 5.7 mg/L)	Brain	No association with average nitrate level
	Incidence				
	Rhein-Neckar-Odenwald area	1987–1988			
Van Loon et al. 1998 Netherlands	Prospective cohort		Nitrate intake from public supplies in 1986 and intake of tap water (quintiles; mean level in highest quintile: 3.7 mg/day)	Stomach	No association with quintiles of water nitrate intake (highest quintile: RR = 0.88)
	Incidence	1986–1992			
Ward et al. 1996 USA	Population-based case–control		Average nitrate level in public water supplies 1945–early 1980s categorized into quartiles (lowest: < 1.6; highest: ≥ 4.0 mg/L); Ever exposure ≥ 10 mg/L	Non-Hodgkin lymphoma	Significant positive trend with increasing quartiles: OR highest quartile = 2.0 (95% CI, 1.1–3.6)
	Incidence				
	66 counties in eastern Nebraska	1983–1986			
Ward et al. 2003 USA	Population-based case–control		Average nitrate level in public water supplies 1960–1987 (highest quartile men: 3.1 mg/L; women: 2.4 mg/L); Years of exposure ≥ 10 mg/L	Bladder	Inverse association with quartiles of average level among men; no association among women. Similar results for years ≥ 10 mg/L
	Incidence				
	Iowa	1986–1989			
Ward et al. 2004 USA	Population-based case–control		Average nitrate level in public water supplies	Brain (gliomas)	No association with quartiles of the average nitrate level
	Incidence	1988–1993	1960–1986		
	66 counties of eastern Nebraska				
Weyer et al. 2001 USA	Prospective cohort		Average nitrate level (1955–1988) in public water supplies for residence at enrollment (highest quartile: > 2.46 mg/L)	Non-Hodgkin lymphoma, leukemia, colon, rectum, pancreas, kidney, bladder, breast, ovary, uterine corpus, lung and bronchus, melanoma	Positive associations with average nitrate level for bladder (highest quartile OR = 2.83) and ovary (OR = 1.84) and inverse associations for uterus (highest quartile OR = 0.55) and rectal cancer (OR = 0.47)
	Incidence	1986–1998			
	Iowa				

OR, odds ratio.

a Nitrate levels presented in the original publications as mg/L nitrate were converted to mg/L nitrate-N.

**Table 2:** Studies of the relation between drinking-water nitrate^a^ and reproductive outcomes.

Reference, study population, study design	Measurement of water nitrate	Reproductive outcome	Reported findings
Aschengrau et al. 1989 Massachusetts (USA) residents Hospital case–control study	Matched maternal residence at pregnancy outcome to results of tap water samples	SBs through 27 weeks of gestation	OR of 0.5 for SB with exposure to water nitrate levels of 0.1–5.5 mg/L relative to nondetectable levels
Grant et al. 1996 Indiana (USA) Cluster investigation	Wells tested for nitrates after cluster reported	SBs	Water nitrate above U.S. EPA MCL for women with SBs
Aschengrau et al. 1993 Massachusetts (USA) residents Hospital case–control study	Matched maternal residence during pregnancy or outcome to results of tap water samples	Congenital anomalies, stillbirths, neonatal deaths	Neither stillbirths nor congenital anomalies associated with detectable levels of water nitrate (0.2–4.5 mg/L); small positive association between water nitrates and neonatal deaths.
Super et al. 1981 South West Africa Cross-sectional study	Water sample taken from well used at time of home visit	Spontaneous premature labor Size of infant at birth	No association between water from high nitrate regions and prematurity or size of infant
Bukowski et al. 2001 Prince Edward Island, Canada Case–control study	Residential postal code at time of delivery linked to nitrate level exposure map	IUGR Premature birth	Dose–response relation between nitrate level and ORs for IUGR and prematurity
Scragg et al. 1982 Dorsch et al. 1984 South Australia Case–control study	Address at delivery linked to sources of water and data on nitrates	Congenital malformations	Elevated OR for any congenital malformation (2.8); malformations of the CNS (3.5); musculoskeletal system (2.9) if primarily drank groundwater. Elevated ORs for congenital malformations associated with nitrate levels ≥ 5 mg/L relative to nitrate levels < 5 mg/L
Arbuckle et al. 1988 New Brunswick, Canada Case–control study	Collected and analyzed a water sample at maternal residence at time of index birth	Congenital malformations of the CNS	OR of 2.3 for CNS malformations with exposure to nitrate 26 mg/L relative to baseline of 0.1 mg/L
Ericson et al. 1988 All deliveries in Sweden Case-control study	Earliest known maternal address linked to water nitrate results	NTDs	Average water nitrate similar between cases and controls
Croen et al. 2001 California (USA) Case–control study	Linked periconceptional addresses to water companies and databases	NTDs	Exposure to water nitrates > 45 mg/L associated with anencephaly (OR 4.0) but not with spina bifida; increased risks for anencephaly at water nitrate levels below U.S. EPA MCL among groundwater drinkers only; dietary nitrate and nitrite not associated with NTDs
Cedergren et al. 2002 Ostergotland County, Sweden Retrospective cohort study	Linked periconceptional or early pregnancy address to water supplies using a geographic information system	Any congenital cardiac defect	Weak association (OR 1.2) between water nitrate ≥ 2 mg/L and cardiac malformations
Brender et al. 2004a Brender et al. 2004b Texas (USA) Counties along Texas–Mexico border Case–control study	Usual periconceptional drinking-water source tested for nitrates	NTDs	OR of 1.9 if water nitrates ≥ 3.52 mg/L; increased water nitrate associated with spina bifida (OR 7.8) but not with anencephaly (OR 1.0); slightly inverse relation between dietary nitrite, total nitrite intake and NTDs

Abbreviations: CNS, central nervous system; IUGR, intrauterine growth retardation; SB, spontaneous abortion.

a Nitrate units are mg/L as nitrate.
